# Estrogen-Related Receptor Alpha Modulates Lactate Dehydrogenase Activity in Thyroid Tumors

**DOI:** 10.1371/journal.pone.0058683

**Published:** 2013-03-13

**Authors:** Delphine Mirebeau-Prunier, Soazig Le Pennec, Caroline Jacques, Jean-Fred Fontaine, Naig Gueguen, Nathalie Boutet-Bouzamondo, Audrey Donnart, Yves Malthièry, Frédérique Savagner

**Affiliations:** 1 UMR694, INSERM, Angers, France; 2 Université d’Angers, Angers, France; 3 Département de Biochimie Génétique, CHU d’Angers, Angers, France; 4 Max Delbrück Center for Molecular Medicine, Berlin, Germany; 5 UMR915, INSERM, Nantes, France; University of South Alabama, United States of America

## Abstract

Metabolic modifications of tumor cells are hallmarks of cancer. They exhibit an altered metabolism that allows them to sustain higher proliferation rates in hostile environment outside the cell. In thyroid tumors, the expression of the estrogen-related receptor α (ERRα), a major factor of metabolic adaptation, is closely related to the oxidative metabolism and the proliferative status of the cells. To elucidate the role played by ERRα in the glycolytic adaptation of tumor cells, we focused on the regulation of lactate dehydrogenases A and B (LDHA, LDHB) and the LDHA/LDHB ratio. Our study included tissue samples from 10 classical and 10 oncocytic variants of follicular thyroid tumors and 10 normal thyroid tissues, as well as samples from three human thyroid tumor cell lines: FTC-133, XTC.UC1 and RO82W-1. We identified multiple cis-acting promoter elements for ERRα, in both the *LDHA* and *LDHB* genes. The interaction between ERRα and LDH promoters was confirmed by chromatin immunoprecipitation assays and *in vitro* analysis for LDHB. Using knock-in and knock-out cellular models, we found an inverse correlation between ERRα expression and LDH activity. This suggests that thyroid tumor cells may reprogram their metabolic pathways through the up-regulation of ERRα by a process distinct from that proposed by the recently revisited Warburg hypothesis.

## Introduction

The estrogen-related receptor alpha (ERRα) is an orphan nuclear receptor involved in the regulation of mitochondrial biogenesis through the oxidation of fats and glucose [1]–[3]. Recently, ERRα has also been considered as a switch regulating not only the mitochondrial function but also glycolysis so as to maintain a steady level of ATP production, particularly when mitochondrial biogenesis is decreased [4]–[5]. ERRα binds to the ERR response element (ERRE) leading to the regulation of the cellular energy metabolism according to endogenous or exogenous stimuli [2], [6], [7]. This transcription factor may interfere with the three transcriptional coactivators of the PGC-1 family, i.e. the PPARγ coactivator-1α (PGC-1α), the PPARγ coactivator-1β (PGC-1β) and the PGC-1-related coactivator (PRC), all of which serve as mediators between the environment and the transcriptional machinery. PGC-1α and PGC-1β are mainly associated with the modulation of metabolic pathways in tissues that require high oxidative energy production, such as the heart and skeletal muscle [8]. Unlike PGC-1α and PGC-1β, PRC is ubiquitous and more abundantly expressed in proliferating cells. Recent report on deficient PRC mice underlines the non redundant role for this coactivator related to others members of the family [9]. We have shown that the ERRα-PRC transcriptional complex plays a consistent role in thyroid proliferative cells by increasing the coupling efficiency of mitochondria in oxidative cells, and through some other pathway in glycolytic cells [6]. The implication of PRC-ERRα complex in the direct regulation of key enzymes of the glycolytic pathway, such as lactate deshydrogenase (LDH), needs to be investigated.

LDH is a tetrameric enzyme composed of two subunits, M and H, encoded by the LDHA and LDHB genes respectively. Each subunit has specific kinetic properties with LDHA being usually associated with pyruvate-to-lactate conversion, and LDHB with lactate-to-pyruvate conversion [10], [11]. The combination of subunits results in five isozymes (A4, A3B1, A2B2, A1B3, and B4) with tissue-specific distribution [10]: the isoenzymes containing large proportions of LDHB tend to predominate in tissues with aerobic metabolism (e.g. heart) while those containing mainly LDHA are found in tissues with considerable anaerobic metabolism (e.g. skeletal muscle and liver). In addition, the ratio LDHA/LDHB may have significant physiological effects on the isoenzyme pattern. The level of LDHA is elevated in many cancers and plays a crucial part in tumor progression, but the link between invasive tumor development and glycolysis is poorly understood. The role of LDHB in tumor development is less well characterized [12]. Down-regulation of LDHB has a greater effect on lactate production than the induction of LDHA [13], [14]. The modulation of the expression of LDHB could maintain the mitochondrial defect that contributes to the invasiveness of cancer. Moreover, LDHB has been identified as a direct downstream target of the PI3K/AKT/mTOR pathway and should be considered as a therapeutic target of interest for tumors with a high potential of invasiveness [15].

Considering the crucial role of ERRα and the PGC-coactivator family in the regulation of metabolic pathways, their implication in the metabolic switch often associated with tumor progression needs to be investigated. We studied 30 thyroid tumors and three human thyroid tumor cell lines, i.e. FTC-133, XTC.UC1 and RO82W-1, to investigate the role of ERRα in the integrative regulation of the glycolytic metabolism and cell proliferative status.

## Materials and Methods

### Tissue Samples

The study was approved by the ethics committee at the University Hospital of Angers (France), and all patients gave written informed consent. Samples, rendered anonymous before beginning the study, consisted of 10 classical follicular thyroid tumors, 10 oncocytic variant of thyroid tumors and 10 normal thyroid tissues. All samples were obtained from the Ambroise Paré Hospital (Paris, France).

### Cell Cultures

Three human follicular thyroid carcinoma cell lines were used: the XTC.UC1 cells were oncocytic variants kindly provided by O. Clark [16], and the other cell lines, FTC-133 and RO82 W-1, were obtained from the Interlab Cell Line Collection (National Institute for Cancer Research, Genoa, Italy) and originated from classical follicular carcinomas.

FTC-133 and XTC.UC1 cells were grown in Dulbecco’s modified medium (Invitrogen Corp., Carlsbad, CA, USA), supplemented with 10% fetal bovine serum (Seromed, Biochrom AG, Berlin, Germany), 1% L-glutamine (Invitrogen, Carlsbad, CA, USA) and 1% penicillin/streptomycin (Invitrogen, Carlsbad, CA, USA). We added 10 mU/ml TSH (Sigma-Aldrich, Saint Louis, MO, USA) for XTC.UC1.

RO82 W-1 cells were grown in 60% Dulbecco’s modified medium, and 30% endothelial basal medium (both from PAA, Pasching, Austria) supplemented with 10% fetal bovine, 1% L-glutamine, and 1% penicillin/streptomycin.

For treatment with the inverse agonist XCT790 (Sigma-Aldrich, Saint Louis, MO, USA) was used a concentration validated for its specific ERRα inhibition in our cellular models [6]. FTC-133 and RO82W-1 cells were treated for 10 days with a final concentration of 5 µM XCT790, replaced with fresh media every three days.

### Bioinformatics Analysis of LDH Promoters

We extracted LDHA and LDHB promoter sequences from nucleotides −2000 to −1 starting from the transcription starting site (TSS) according to the NCBI accession NM_00566 and NM_002300. We scanned the promoters with the Matrix-Scan software (http://rsat.ulb.ac.be/rsat/) using a position-weight matrix defining ERRα binding sites as described elsewhere [17]. The transcription-factor binding site representations were considered statistically significant at 5% risk after simultaneous comparison with a set of 100 human promoters.

### ERRα Chromatin Immunoprecipitation (ChIP)

ERRα-ChIP assays were performed on 10^6^ XTC.UC1 cells/assay using an anti-human ERRα antibody (sc-65714 from Santa Cruz, CA, USA) according to the protocol provided by the manufacturer (EZ-ChIP, Upstate, Millipore, Billerica, MA, USA). A rabbit anti-goat IgG (55335, MP Biomedicals, CA, USA) was used as a control of non-specific immunoprecipitation. Pellets were dissolved in water and quantitative PCR reactions were performed on the immunoprecipitates and the input DNA using the following primers designed for *LDHA* and *LDHB* genes:

LDHA: 5′-TTGAAGGGAGAGATGATGGA-3′ and 5′-CCAGCCGTGATAATGACCAG-3′

LDHB: 5′-TGCTCTTGTGGATGTTTTGG-3′ and 5′-CTCTCCCCTTCTTGCTGACG-3′

ChIP was considered positive when the gene expression was enriched at least 3-fold in the IgG fraction following the 2^−ΔΔCT^ method [18].

### Cloning of the Human LDHB Promoter and Construction of the Reporter Plasmids

*LDHB* promoter reporter plasmids were constructed using a human genomic DNA fragment from the 5′-flanking region of the human LDHB promoter to the TSS as matrix. To generate the different constructions of the reporter plasmids p.LDHB-Luc 1188, p.LDHB-Luc 611, p.LDHB-Luc 515 and p.LDHB-Luc 105, we amplified the *LDHB* promoter using the same reverse primer (5′-AAGCTTCTACCAGGAGAGAGAAGGCT-3′) and forward primers as follows:

p.LDHB-Luc 1188∶ 5′-AGATCTGGCACTGAGAATAAACTGAA-3′,

p.LDHB-Luc 611∶ 5′-AGATCTCTGTAATCCCAGCACTTTGG-3′,

p.LDHB-Luc 515∶ 5′-AGATCTCCCCTCTCTACTAAAAATAC-3′,

p.LDHB-Luc 105∶ 5′-AGATCTTGAAGGGGATTGAGCGAG-3′.

PCR products were doubly digested with Bgl2 and HindIII and inserted into the pGL3-basic vector. The identity of the constructions was confirmed by sequencing.

### Transient Transfections and Luciferase Assay

RO82W-1 cells were plated two days before transfection. Transient transfection was performed with lipofectamine (Invitrogen, Carlsbad, CA, USA) as recommended by the manufacturer. Cells were collected 48 h later for functional and quantitative PCR analyses.

According to the experiments, RO82W-1 cells were transfected with 1 µg LDHB promoter reporter plasmid (p.LDHB Luc), 0.05 µg of plasmid PRC (Origene Technologies, Rockville, MD, USA), 0.05 µg of plasmid ERRα (Addgene, Cambridge, MA, USA) and 0.5 µg of pRL-CMV (Promega, Madison, WI, USA) used as an internal control of transfection efficiency.

For experimentation with luciferase activity, cells were harvested after 48 h of treatment for the luciferase reporter assay using the Dual-Luciferase Reporter Assay System (Promega). Luciferase activity was normalized to that of the internal control, Renilla luciferase, used as the relative luciferase unit. All assays were done in duplicate in three separate experiments.

### siRNA

To knock down ERRα expression in FTC-133 cells, we transfected predesigned ERRα siRNAs (s4830) and scrambled control siRNA (AM4635) with siPORT NeoFX. After 48 h, cells were harvested for assays. All cells were tested for the down-expression of ERRα, and siRNA was considered efficient when the ERRα expression was inhibited by at least 70% compared to scramble control.

### Western Blot

Cells were rinsed in PBS, trypsinized and collected in centrifuge tubes. Proteins (20µg) were separated by SDS-PAGE and transferred to polyvinylidene difluoride membranes (Hybond-P, Amersham International plc, Little Chalfont, UK) by electroblotting. The membranes were incubated in 5% non-fat milk in TBS-Tween (tris-buffered saline (TBS) with 0.1% Tween-20). The membranes were incubated overnight with dilutions (1/2000) of the following antibodies: monoclonal anti- β-Actin, anti-LDHA, anti-LDHB and anti-ERRα (all obtained from Abcam, Cambridge, UK). After several washes in TBS-Tween, the membranes were incubated with an appropriate chemiluminescent-labelled horseradish peroxidase-conjugated secondary antibody (Jackson ImmunoResearch, WestGrove, PA, USA). The blots were developed using the enhanced chemiluminescence method (ECLplus, Amersham Pharmacia Biotech, Buckinghamshire, UK). Signal quantification was performed by non-saturating picture scanning by a gel Doc 1000 Molecular Analyst apparatus (Biorad, Hercules, CA, USA).

### Quantitative RT-PCR Analyses

Total RNA was isolated using the RNeasy kit (Qiagen, Hilden, Germany) for cultured cells, and trizol procedure for thyroid tissues (Invitrogen).

Reverse transcription was performed on 1 µg of RNA with Advantage RT-for-PCR kit (Clontech, Palo Alto, CA, USA) following the manufacturer’s recommendations.

Real-time quantification was performed in a 96-well plate using the IQ SYBR Green SuperMix and Chromo4 (Biorad). Data were normalized to β-globin as described elsewhere [6].

### Respiratory Parameters

Respiratory parameters were investigated on intact cells from cultured cell lines and sample tissues by polarography, using a high-resolution Oroboros O2k oxygraph (Oroboros Instruments, Innsbruck, Austria) as described elsewhere [19], [20]. The basal respiratory rate, defined as respiration in the cell-culture medium without additional substrates or effectors, was determined by measuring the linear rate of oxygen flux in intact cells (3.10^6^ cells placed at 37°C in 2 ml Dulbecco’s modified medium).

### Enzymatic Activities

The activity of citrate synthase (CS), and Lactate Deshydrogenase (LDH) was measured spectrophotometrically (at 412 nm for CS and 340 nm for LDH) on cell lysates at 37°C in a cell buffer (250 mM saccharose, 20 mM tris[hydroxymethyl]aminomethane, 2 mM EGTA, 1 mg/ml bovine serum albumin, pH 7.2) using a Beckman DU 640 spectrophotometer (Beckman Coulter). Specific enzymatic activities were expressed in mIU (i.e. nanomoles of 5,5-dithiobis(2-nitrobenzoic acid)/min/mg portein for CS or nanomoles of NADH/min/mg protein for LDH). The cellular protein content was determined using the Bicinchoninic assay kit (Uptima, Interchim, Montluçon, France) with bovine serum albumin as standard (All from Sigma-Aldrich, Saint Louis, MO, USA, except Tris from Eurobio, Les Ulis, France). Results were expressed as relative LDH to CS activities as an indicator of global cell metabolism.

Lactate concentration in the culture media was determined by spectrophotometry using appropriate enzymatic kits (Boehringer Mannheim, Germany) on a Hitachi-Roche 917 (Roche Diagnostics GmbH Mannheim, Germany) and normalized to total cell numbers.

### Microarray Analysis

cDNA from RO82W-1 cells were hybridized in duplicate on human 4×44,000 expression chips (Agilent Technologies, Santa Clara, CA, USA) according to the manufacturer’s recommendations. Data are available in the GEO database (GSE 37017). The Expression Analysis Systematic Explorer (EASE) and Gene Set analysis were used to determine the statistically over-represented and differentially expressed genes. Gene ontology enrichments in gene lists were searched for by means of the GOMiner. The most abundant gene ontology terms, representing at least 5% of the genes in the lists, with p values lower than 0.05, were considered for interpretation.

### Statistical Analysis

Results were expressed as mean values ± standard deviation (SD). The statistical significance of observed variations was assessed using the ***Mann-Whitney test***. Differences were considered significant at p<0.05. All analyses were performed using StatView version 5.0 (SAS Institute, Cary, NC, USA).

## Results

### Expression of ERRα and LDH Genes in Human Thyroid Tissues and Cell Lines

We studied the expression of ERRα and LDH in 30 normal or tumoral human thyroid tissues and three human thyroid cancer cell lines: RO82W-1, FTC-133, XTC.UC1.

**Figure 1:**
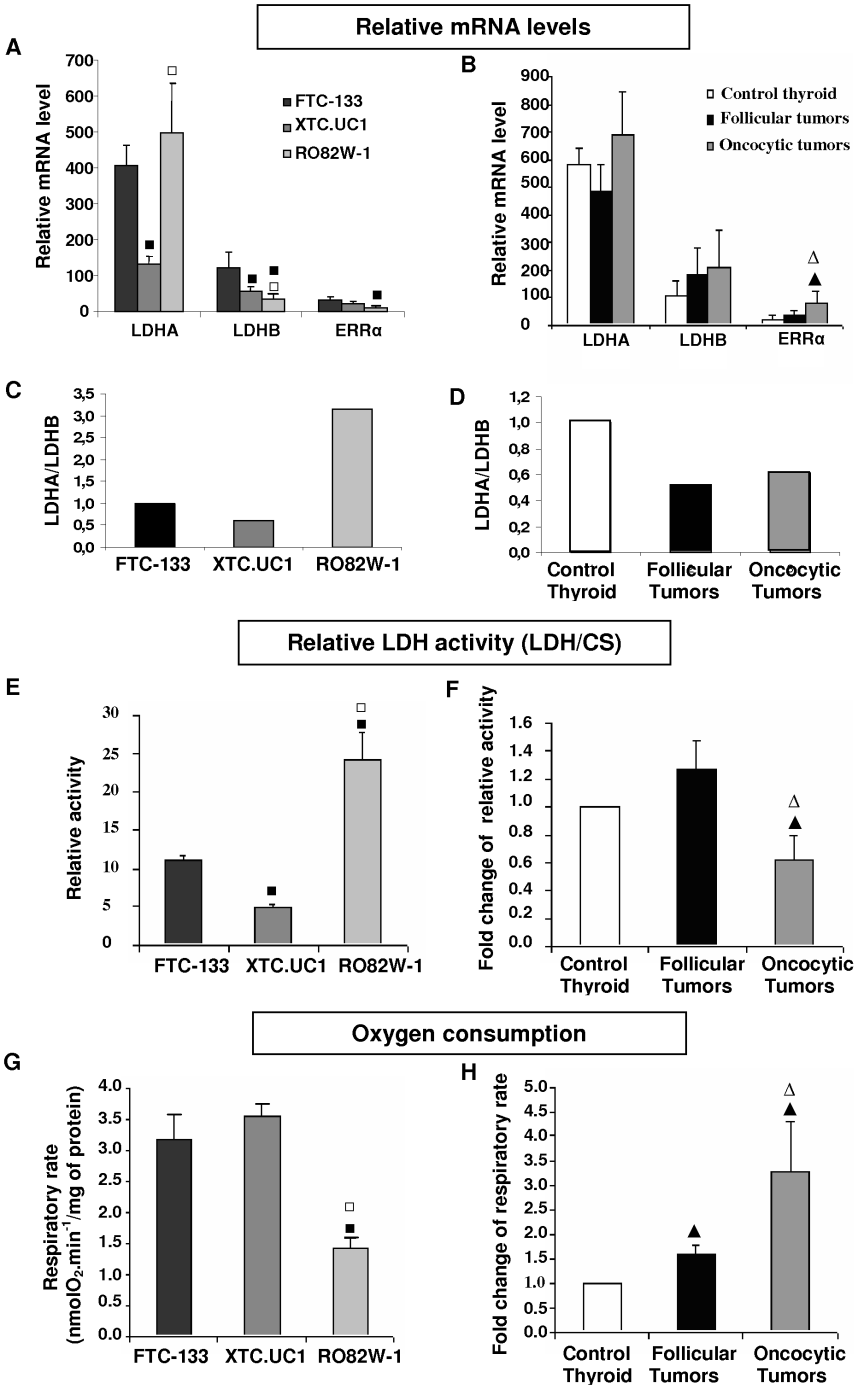
Metabolic analysis for the three cell lines: FTC-133, XTC.UC1, RO82W-1, and 30 thyroid tissues (10 controls, 10 follicular tumors and 10 oncocytic tumors). Relative expression levels of *LDHA*, *LDHB* and *ERRα* determined by quantitative PCR and normalized to *β-globin* for cell lines (A) and thyroid tissues (B). Ratio of expression level of LDHA to LDHB for cell lines relative to FTC-133 (C) and for thyroid tissues relative to control tissues (D); Ratio of LDH to CS activities for cell lines (E) and thyroid tissues (F); Measurement of oxygen consumption under basal respiratory conditions for cell lines (G) and thyroid tissues (H). Results are the mean values±SD of three experiments for the cell lines and mean values of thyroid tumors relative to control thyroid tissues. ▪ p<0.05 for FTC-133 *versus* XTC.UC1 or RO82W-1, □ p<0.05 for XTC.UC1 *versus* RO82W-1, ▴ p<0.05 for control thyroid *versus* follicular tumors or oncocytic tumors; Δ p<0.05 for follicular tumors *versus* oncocytic tumors.

The thyroid tissues and cell lines expressed the same LDH gene profiles with higher copy numbers of *LDHA* than those of *LDHB* (Figure 1A). The expression level of *LDHA* was four times higher in FTC-133 and RO82W-1 cells than in XTC.UC1 cells. The expression levels of *LDHB* were higher by factors of 3.56 in FTC-133 cells and 1.60 in XTC.UC1 cells, compared to RO82W-1 cells. These results were confirmed at protein levels for LDHA, LDHB (Figure S1). In thyroid tissues, the *LDHA* and *LDHB* expression levels were similar in follicular, oncocytic tumors and normal tissues (Figure 1B). The correlation between protein and mRNA levels for *LDHA* and *LDHB* was less relevant, but related to previous studies on cell metabolism where a mix between aerobic and anaerobic glycolysis was described in normal and tumor cells [21]–[22] (Figure S1).

Using the LDHA/LDHB expression ratio as an indicator of the combined influence of the isoforms on LDH activity, we showed that this ratio was higher for RO82W-1 cells than for FTC-133, or XTC.UC1 cells (Figure 1C). In thyroid tissues the ratio was higher for normal thyroid tissues than for follicular tumors or oncocytic tumors (Figure 1D).Thus, the *LDHA/LDHB* ratio was similar in follicular and oncocytic tumors and their corresponding cell line models, FTC-133 and XTC.UC1.

We evaluated the glycolytic metabolism by lactate dehydrogenase activity (Figures 1E and 1F) and mitochondrial oxygen consumption (Figures 1G and 1H). The relative LDH activity was higher in RO82W-1 cells in which the oxygen consumption rate was lowest. In contrast, the relative LDH activity of FTC-133 and XTC.UC1 cells was 2 to 5 times lower than that of RO82W-1 cells, and the oxygen consumption rates were twice as high. These findings suggest that FTC-133 and XTC.UC1 cells mainly use an oxidative metabolism whereas RO82W-1 cells mainly depend on anaerobic glycolysis. These results were supported by the measurement of total lactate production in the culture media for the three cell lines (Figure S1). In thyroid tissues, the relative LDH activity was similar in normal thyroid and follicular tumors whereas it was 40% lower in oncocytic tumors. Oxygen consumption was 1.58 to 3.25 times higher in follicular and oncocytic tumors, respectively, compared to control thyroid tissues. Thus, oxidative metabolism appeared to be favored in follicular and oncocytic thyroid tumors.

In all thyroid tissues and cell lines, the expression levels of *ERRα* were overexpressed in samples with pronounced oxidative metabolism (Figure 1A and 1B). *ERRα* levels were respectively 1.6 and 4 times higher in follicular and oncocytic tumors compared to normal tissues. Similarly, *ERRα* levels were higher in FTC-133 and XTC.UC1 cells than in RO82W-1 cells. All these results were confirmed at the protein level (Figure S1).

### ERRα Motifs in *LDHA* and *LDHB* Promoters

**Figure 2:**
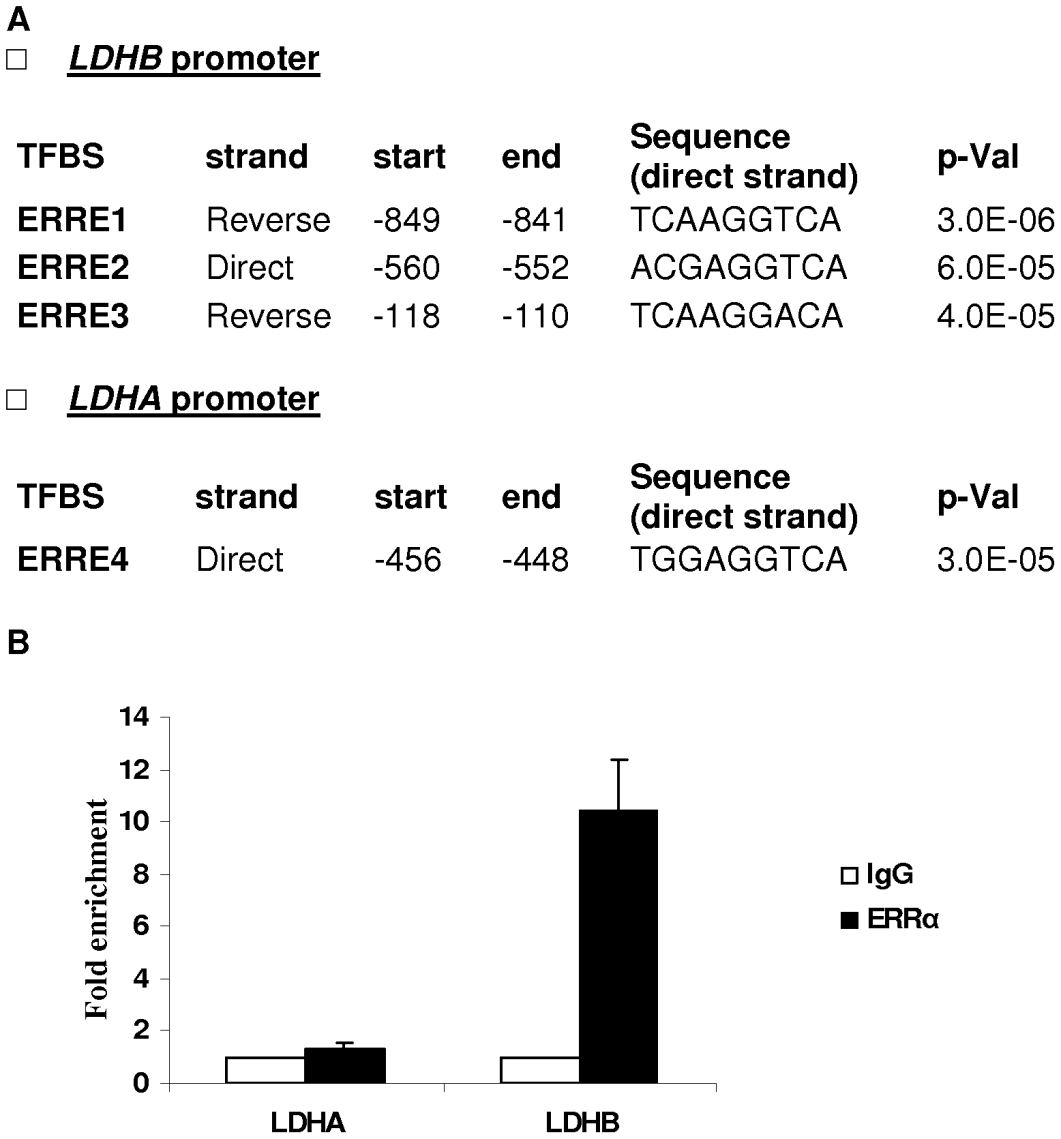
Potential ERRα response elements in *LDHB* and *LDHA* promoters (A) Potential ERRα binding sites numbered relative to the transcription starting site (TSS) (B) Chromatin ImmunoPrecipitation (ChIP) assay for *LDH* promoters in XTC.UC1 cells using a polyclonal ERRα antibody. Chromatin was immunoprecipitated with the indicated antibody and submitted to quantitative PCR. Results are expressed as fold change of enrichment compared to control IgG immunoprecipitated material. ERRα-IP was realized in duplicate and each sample was tested in triplicate for quantitative PCR. TFBS: transcription factor binding site.

We examined the *LDHA* and *LDHB* promoters using ERRα binding-site matrices described elsewhere [17]. The promoter sequences, extracted from nucleotides −2000 to −1, starting from the transcription starting site (TSS), were scanned for ERRα binding sites. The bioinformatics search suggested that one region between −456/−448 for the *LDHA* promoter, and the three regions between −849/−841, −560/−552 and −118/−110 for the *LDHB* promoter, were potential ERRα binding sites (p≤0.05) (Figure 2A).

We confirmed the physical association between ERRα and the *LDHB* promoter in oncocytic cells (XTC.UC1) using the chromatin immunoprecipitation (ChIP) assay with a polyclonal ERRα antibody. We chose these cells because of their high expression levels of ERRα (Figure 1A). A 12-fold enrichment of the *LDHB* gene expression was observed compared to that of the IgG fraction. No enrichment was observed on the *LDHA* promoter (Figure 2B). These findings indicate that ERRα directly interacts with the *LDHB* promoter but not that of *LDHA*.

### Regulation of the *LDHB* Promoter by ERRα

**Figure 3:**
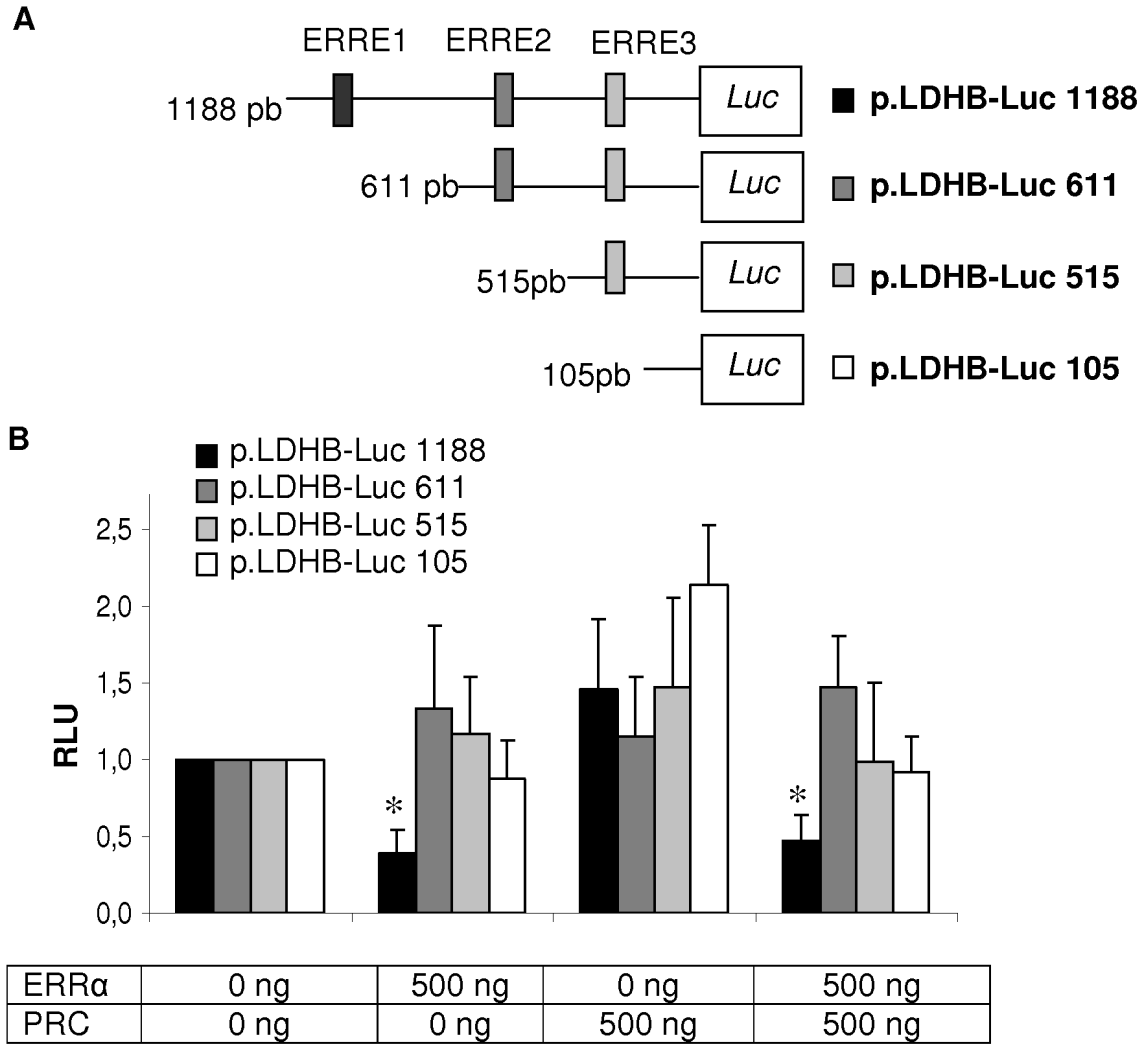
ERRα inhibits *LDHB* promoter activity. (A) Different construction of the human LDHB promoter reporter plasmid. (B) RO82W-1 cells were transfected with the indicated promoter constructs together with the expression plasmid of ERRα and/or PRC. Luciferase activity was determined 48 h after transfection and normalized against renilla luciferase activity. Results, presented in Relative Light Units (RLU), are the mean values±SD of three experiments performed in duplicate. *: p≤0.05 versus cells transfected with plasmids controls and no ERRα or PRC.

To investigate the molecular mechanism of the transcriptional activation of *LDHB* by ERRα, we built four constructions of a genomic DNA fragment from the 5′-flanking region of the human *LDHB* gene into a luciferase plasmid (Figure 3A). We transfected one of these four constructions with ERRα and/or PRC in RO82W-1 thyroid cancer cells, which have low mitochondrial mass and poor expression of ERRα and PRC (Figure 3B). An overexpression of ERRα induced a 60% decrease in transcriptional activity with p.LDHB-Luc 1188 which contains the three potential ERREs. No change was observed with the three other constructions, suggesting that only the ERRE1 (−849/−841 region) is functional. Our earlier work indicated that PRC could be a key partner in the action of ERRα [6], [17]. We therefore transfected PRC, either alone or with ERRα, into each of the four constructions of the *LDHB* promoter. No effect on reporter activity was observed after transfection with PRC alone. The forced overexpression of ERRα and PRC reduced luciferase activity by 53% with the longest *LDHB* promoter but no interaction was observed using the other promoters.

These results suggest that ERRα interacted with the −849/−841 region of the *LDHB* promoter and exerted a negative transcriptional activity. The action of PRC in our model on the LDHB promoter is either null or as a transcriptional coactivator of ERRα.

### ERRα Modulates LDH Activity

**Figure 4:**
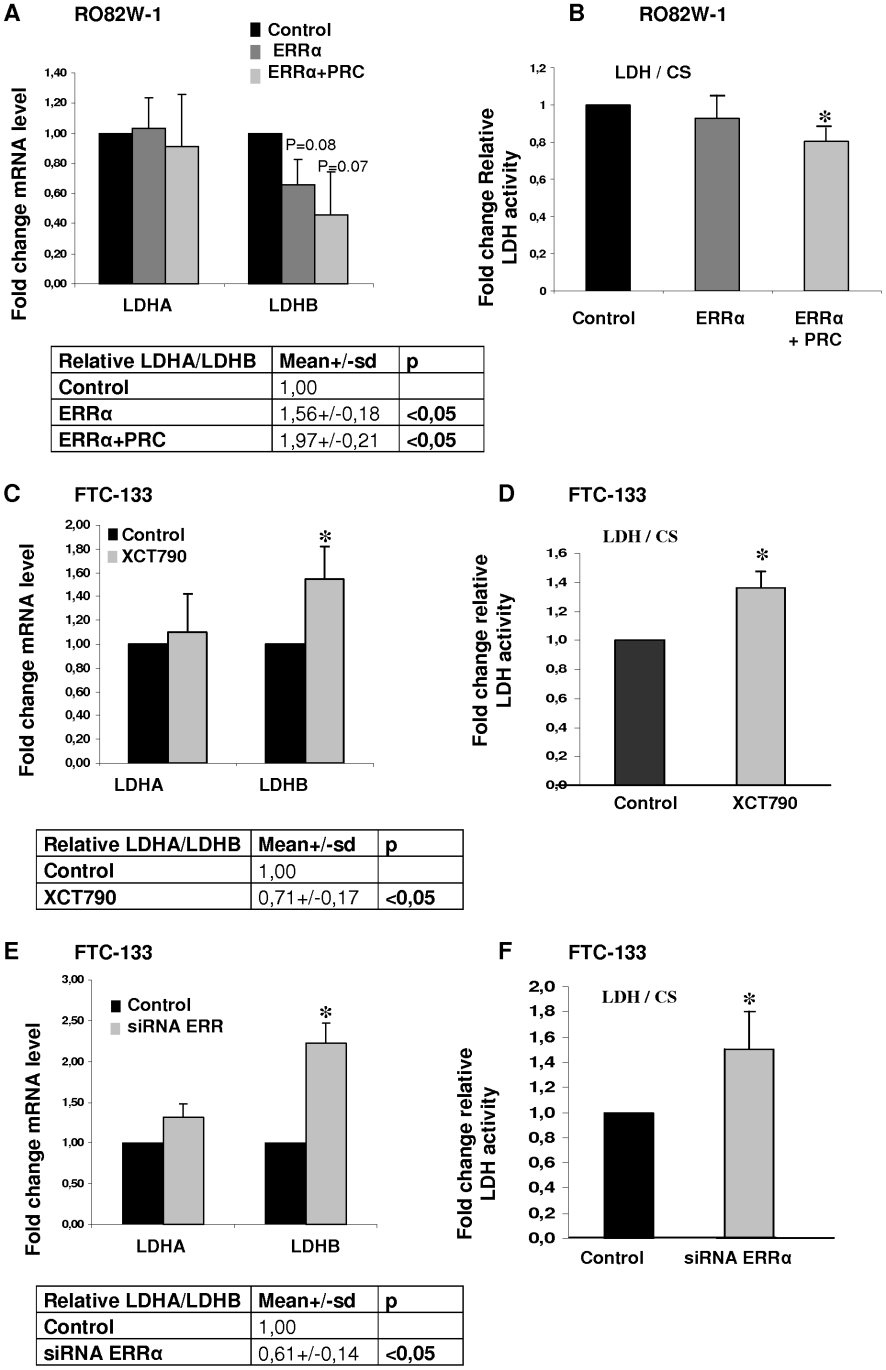
ERRα modulates expression and activity of LDH. *LDHA* and *LDHB* expression levels were measured by quantitative real-time PCR. The ratio of LDH activity to CS activity was determined under various conditions. Measurements were made 48 h after transfection or 10 days of treatment with XCT790 and results are presented relative to the control which was assigned a unit value. *LDHA* and *LDHB* expression levels, the mean *LDHA/LDHB* ratio (A) and the relative LDH activity (B) for RO82W-1 cells transfected with 50 ng ERRα or 50 ng ERRα and 50 ng PRC or empty vectors (Control). *LDHA, LDHB* expression levels and mean of the *LDHA/LDHB* ratio (C) and relative LDH activity (D) for FTC-133 cells treated with XCT790 or vehicle (Control). *LDHA, LDHB* expression levels and mean of the *LDHA/LDHB* ratio (E) and relative LDH activity (F) for FTC-133 cells transfected with control or ERRα siRNA. The results are the mean values±SD of three experiments performed in duplicate relative to controls. *: p≤0.05.

To investigate the role of ERRα and PRC in regulating *LDHB* transcription, we overexpressed *ERRα* and/or *PRC* in RO82W-1 cells, which present low expression levels of endogenous *ERRα* and *PRC*. Overexpression of ERRα, either alone or with PRC, consistently decreased the expression of LDHB at mRNA and protein levels whereas it had no effect on LDHA (Figure 4A and Figure S2). Thus, the *LDHA/LDHB* mRNA expression ratios significantly increased by 56% when ERRα was overexpressed (p<0.05) and by 96% when both ERRα and PRC were overexpressed (p<0.05). Only the overexpression of both ERRα and PRC significantly decreased LDH activity (p<0.05) (Fig. 4B).

We investigated the consequences of the inhibition of ERRα and PRC on LDH activity in FTC-133 cells which present the highest expression of endogenous *ERRα* in the three tumor cell lines. The treatment of these cells with XCT790 significantly increased the expression of LDHB at mRNA and protein levels and that of LDH activity by 36% (Figures 4C, 4D and Figure S2). It had no effect on protein and mRNA levels for LDHA (Figures 4C and Figure S2). Thus, the *LDHA/LDHB* expression ratios decreased by 30%. The same results were obtained when we inhibited ERRα with XCT790 and PRC with specific siRNA (data not shown). To exclude the action of XCT790 on other proteins, we confirmed these results by *ERRα* siRNA. The inhibition of ERRα significantly increased the protein and mRNA levels for LDHB but had no effect on protein and mRNA levels for LDHA (Figure 4E and Figure S2); LDH activity increased by 50% (Figure 4F).

Our results show that ERRα regulates LDH activity by direct interaction with the *LDHB* promoter in cellular models of follicular thyroid carcinoma, and that PRC may interact with ERRα to decrease LDH activity and modulate the LDHA/LDHB expression ratio.

### ERRα Coregulates the Cell Cycle and the Metabolism

**Figure 5:**
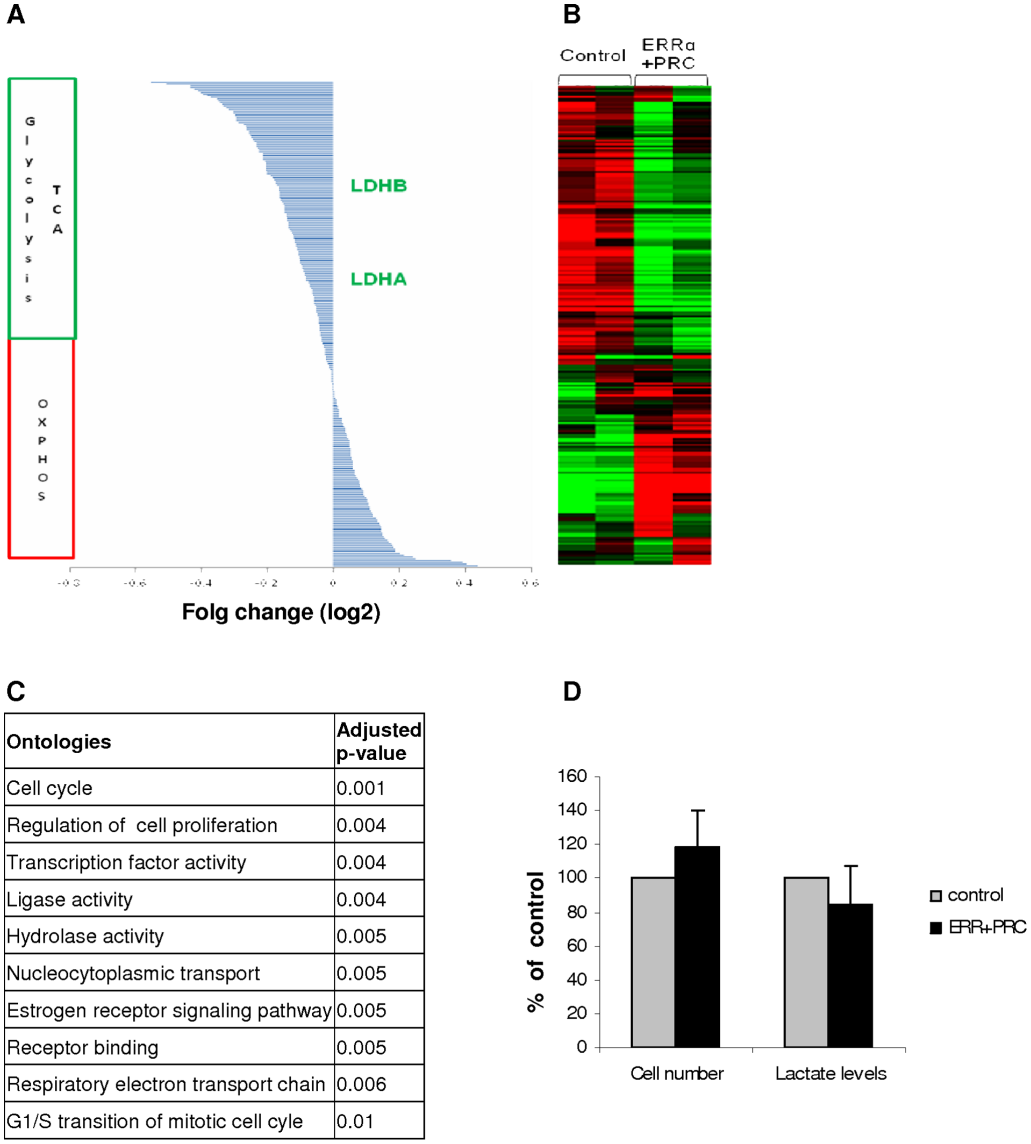
ERRα and PRC coregulate the cell cycle and metabolism in thyroid cells. RO82W-1 cells transfected with ERRα and PRC or empty vector (Control) and analysed 72 h after transfection. Pangenomic microarray results for metabolism genes. Gene-expression levels are grouped by class and ordered according to their mean log level of expression (A) or color-coded in the matrix from green (underexpression) to red (overexpression) (B). Ten main gene ontologies (C). Cell proliferation and lactate levels in media (D).

Since a significant decrease in LDH activity was observed only when combining the overexpressions of PRC and ERRα, we chose to explore differential gene expression by cDNA microarray comparing RO82W-1 cells transfected with both ERRα and PRC, and cells transfected with control plasmids. The pangenomic microarray data showed that the overexpression of ERRα and PRC induced the differential expression of 553 genes (Figure 5A and 5B). The main gene ontology showed a significant overexpression of genes implicated in the cell cycle, regulation of cell proliferation and G1/S transition of the mitotic cell cycle when ERRα and PRC were overexpressed (Figure 5C). Nevertheless, RO82W-1 cells transfected with control plasmids or both PRC and ERRα plasmids during 72 h had the same proliferative statuses (Figure 5D). We found an overexpression of genes implicated in estrogen receptor signaling due to the high degree of sequence identity between ERR and the estrogen receptor. Moreover, we found an overexpression of the respiratory electron transport chain as described previously [6].

Of the 553 genes with differential expressions due to ERRα and PRC, 288 were involved in the metabolism. Among these 288 genes, we found a positive regulation for 40% of the genes of respiratory chain and a negative regulation for 86% of the genes of TCA and anaerobic glycolysis (Figure 5A). We verified that the lactate production was not increased when the cells were transfected with ERRα and PRC (Figure 5D).

## Discussion

The modification of cellular metabolism in tumors has recently been characterized as one of the major alterations of tumor cells [23]. Indeed, tumor cells, which have an altered carbohydrate metabolism, produce ATP from glucose through oxidative phosphorylation (OXPHOS) and anaerobic glycolysis even under normal oxygen pressures. This metabolic strategy offers tumors a selective advantage by satisfying the high ATP demands, facilitating the macromolecular biosynthesis required by rapidly proliferating tumors, and allowing NAD^+^ production in the absence of mitochondrial oxidation.

The regulation of cell metabolism requires the expression of a large number of genes encoded by the nuclear and the mitochondrial genome. The coordination of these genes depends on transcription factors among which ERRα appears to be essential [24]. ERRα is known to coregulate and coordinate gene-encoding enzymes of the biochemical pathways involved in the generation of energy from glucose *via* OXPHOS. Our findings indicate that ERRα may also regulate anaerobic glycolysis *via* LDH activity in thyroid tumors. The Warburg effect having been recently revisited, a more realistic description of cancer cell metabolism suggests that oxidative phosphorylation and anaerobic glycolysis cooperate to sustain energy needs during tumorigenesis [25]. In breast cancer it has been shown that miRNA-378 regulates the metabolic switch *via* the ERRγ-PGC1β complex, which promotes oxidative metabolism, and the ERRα-PGC1β complex, which favors activation of the glycolytic pathway [26]. We have shown that ERRα controls the cell cycle and promotes the efficiency of oxidative phosphorylation by interfering with PRC coactivators and according to the metabolic status of the cells in thyroid tumors [6]. Our study of LDH activity and oxygen consumption shows that, compared to normal tissues and follicular tumors, oncocytic tumors mainly depend on oxidative metabolism. This type of altered metabolism has already been described in other tumors, such as hepatomas, melanomas and lung carcinomas [27], [28]. This oxidative metabolism could be orchestrated by ERRα. Indeed, the expression of the ERRα gene is greater in oncocytic tumors than in normal thyroid tissues and follicular tumors. Hypothesizing that ERRα coregulates OXPHOS and glycolytic pathways in an aerobic environment, we investigated the regulation of the glycolytic enzyme LDH in cellular models of thyroid tumors. We choose cellular models presenting oxidative or glycolytic metabolism, to explore mechanisms for oxidative maintenance in XTC.UC1 and FTC133, and the role of ERRα in RO82W-1 metabolic reversion. Investigating the promoters of the *LDHA* and *LDHB* genes we found responsive elements for ERRα in both genes. However, chromatin-immunoprecipitation studies and experiments involving transient transfection confirmed the functional interaction only for the *LDHB* gene. These results are in accordance with a recent study showing that *LDHB* is regulated by ERRα and Prox1 whereas *LDHA* expression only depends on Prox1. Interestingly, Prox1 has been shown to inhibit the activity of the ERRα-PGC-1α complex in liver cells [29]. In contrast, it has been found that ERRα activates the LDHA gene promoter through ERRE in HepG2 human liver cells or MCF7 human breast cancer cells [4]. This divergent regulation of bioenergetic functions *via* the action of ERRα transcriptional complex highlights the role of the cofactors in the context of proliferative cells.

Lactate dehydrogenase is composed of two subunits, M and H, which enter into five different combinations. The expression levels of LDHA and LDHB expression determine the cellular isozyme pattern [10]. A study based on a mathematical model of the LDH reaction concluded that the decisive parameter was the total LDH activity and not the isoenzyme pattern [30]. The situation is quite different during metabolic transitions in energy metabolism, in particular when the glycolytic flux changes as it does in tumors [31]. In such cases, the LDH isoform ratio may serve as an indicator of the relative flux through the aerobic and anaerobic systems. Indeed, in our study, oncocytic and follicular tumors were more dependent on oxidative metabolism, expressed more ERRα, and had a lower LDHA/LDHB ratio than control thyroid tissues. The results were similar for thyroid cell lines presenting an ERRα expression and an LDHA/LDHB ratio in relation to their metabolic status. We found a functional ERRE on the LDHB promoter that negatively regulated LDHB expression. Surprisingly, in thyroid tissues and cell lines, the expression levels of ERRα and LDHB were correlated. We hypothesised that this coregulation could be orchestrated by mTOR, which is often deregulated in thyroid tumors [32]. Indeed, mTOR has been identified as a positive regulator of ERRα and LDHB [15], [33]. Thus, *ERRα* and *LDHB* expression may be coregulated independently of the potential regulation of *LDHB* by ERRα. Contrary to other studies, the LDHA promoter has no functional ERRE in our cellular models [4]. We postulate that difference in the cofactors (PGC-1alpha/PRC ratio) in our tumor cells may have an impact on the expression of LDH genes. We have shown that our three cell lines expressed 3.6 to 8 times more PRC than PGC1α [6]. PRC and ERRα are known to play a role in the transition from the G1 phase to the S phase of the cell cycle [34], [35], and to regulate the energy needs of the cells. Our study showed an overexpression of genes implicated in the regulation of the cell cycle, with a similar proliferation of cells after 72 h of transfection with ERRα and PRC. We need to confirm these findings by using cell lines with stable transfection of ERRα and PRC and evaluating cell proliferation after a longer period. Our previous work showed that the inhibition of ERRα by XCT790 decreased cell proliferation after 10 days of treatment (6). Thus, the transition and modification of tumor cell proliferation could be orchestrated by the ERRα-PRC complex by two mechanisms: regulation of the cellular energy metabolism and alteration of the LDH subunit composition. The down-expression of *LDHB* could transform the LDH isoenzyme into the LDH5 isoenzyme containing only the M subunits coded by the *LDHA* gene known to be linked to metastatic progression [36], [37]. A similar change in the isoform pattern adapting tumor cells to environmental conditions has been described for hexokinase and phospho-fructokinase, two enzymes of the glycolytic pathway [38], [39]. Moreover, the release of lactate decreases the pH in the extracellular space, destroying adjacent normal cells, degrading the extracellular matrix and facilitating tumor invasion [13]. Our findings suggest that ERRα may influence tumor aggressiveness by metabolic modification and modulation of the LDHA/LDHB expression ratio.

Our observations show that the ERRα transcriptional complex is important in the induction of a metabolic shift in thyroid cell lines. Further experiments are needed to validate ERRα as a key factor of metabolic shift in thyroid tumors event though our results argue in favor of a pivotal role of ERRα for the regulation of both oxidative and glycolytic metabolism for oxydative thyroid tumors.

## Supporting Information

Figure S1 Basal protein levels of LDH, ERRα and lactate production. Quantitative protein level of LDHA, LDHB and ERRα in thyroid cell lines (A) and thyroid tissues (B) were determined by western blot and presented relative to the control (β-Actin) that was assigned a value of unity (n = 2). (C) Total lactate production by thyroid cell lines (n = 3 in triplicate).
(TIF)

Figure S2 ERRα modulates protein levels of LDHB (**A**) Quantitative protein levels of LDHA, LDHB and ERRα were determined for RO82W-1 cells transfected with 50 ng ERRα or 50 ng ERRα and 50 ng PRC or empty vectors (Control). Measurements were made 48 h after transfection by western blot and presented relative to the control (β-Actin) that was assigned a value of unity (n = 2). (**B**) Quantitative protein levels of LDHA, LDHB and ERRα were determined for FTC-133 cells treated for 10 days with XCT790 or vehicle (Control). Measurements were made 48 h after transfection by western blot and presented relative to the control (β-Actin) that was assigned a value of unity (n = 2).
(TIF)
