# Supplementary figures and images for: Estrogen-Related Receptor Alpha Modulates Lactate Dehydrogenase Activity in Thyroid Tumors
Source: PLoS One. 2013 Mar 13;8(3):e58683. doi: 10.1371/journal.pone.0058683 (PMC3596295; doi:10.1371/journal.pone.0058683)

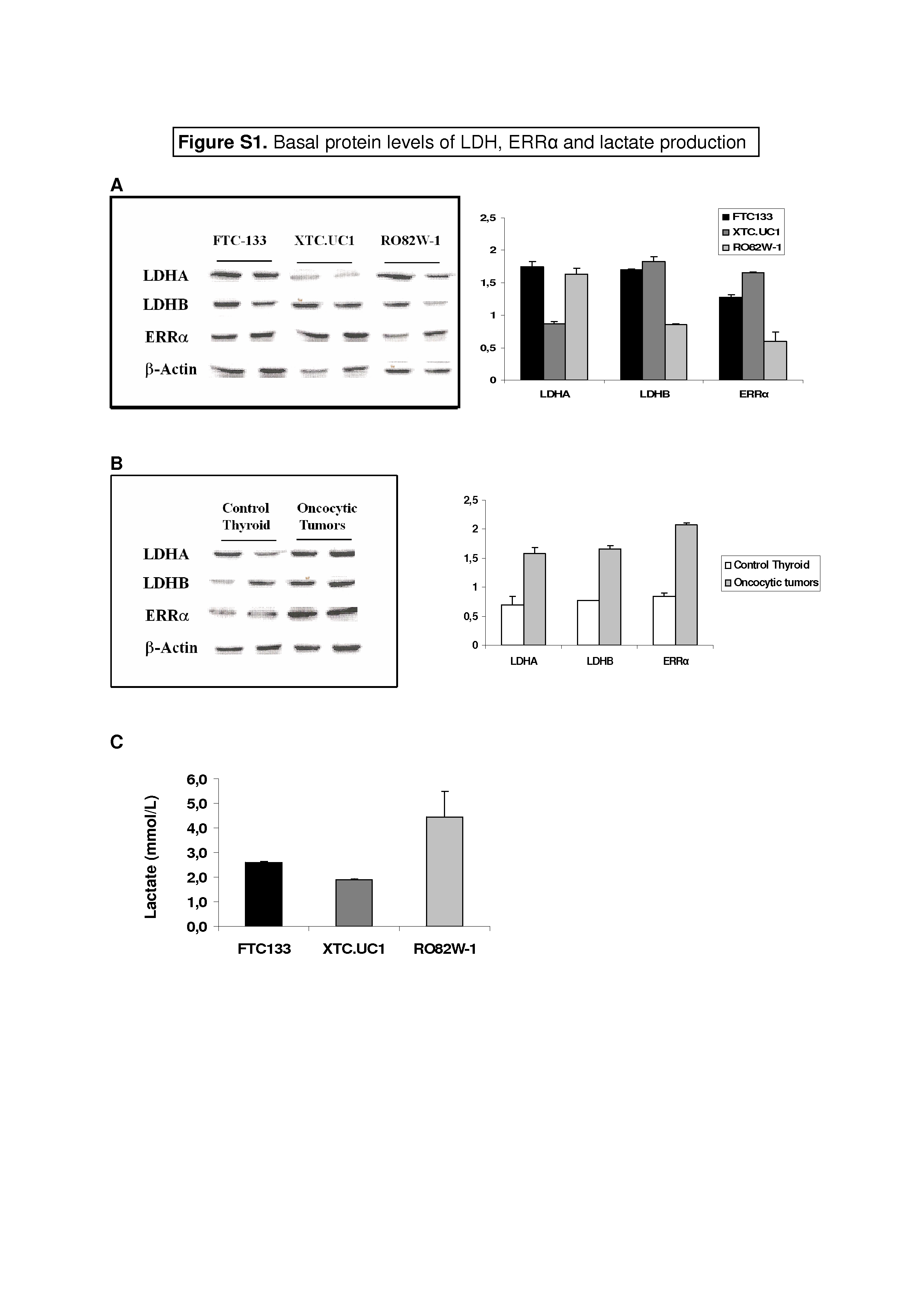

Supplement: Figure S1 — Basal protein levels of LDH, ERRα and lactate production. Quantitative protein level of LDHA, LDHB and ERRα in thyroid cell lines (A) and thyroid tissues (B) were determined by western blot and presented relative to the control (β-Actin) that was assigned a value of unity (n = 2). (C) Total lactate production by thyroid cell lines (n = 3 in triplicate). (TIF) [file pone.0058683.s001.tif]

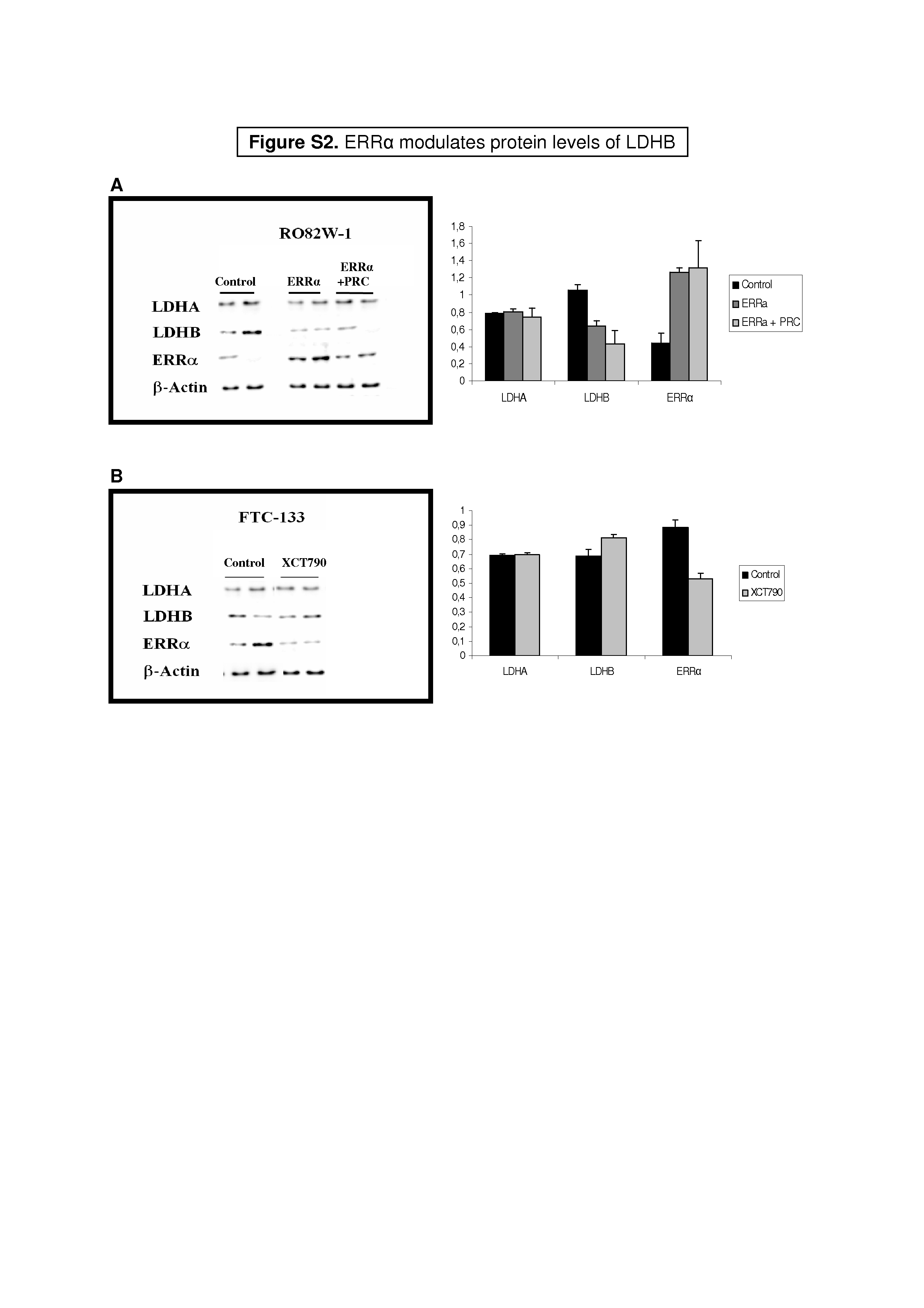

Supplement: Figure S2 — ERRα modulates protein levels of LDHB (A) Quantitative protein levels of LDHA, LDHB and ERRα were determined for RO82W-1 cells transfected with 50 ng ERRα or 50 ng ERRα and 50 ng PRC or empty vectors (Control). Measurements were made 48 h after transfection by western blot and presented relative to the control (β-Actin) that was assigned a value of unity (n = 2). (B) Quantitative protein levels of LDHA, LDHB and ERRα were determined for FTC-133 cells treated for 10 days with XCT790 or vehicle (Control). Measurements were made 48 h after transfection by western blot and presented relative to the control (β-Actin) that was assigned a value of unity (n = 2). (TIF) [file pone.0058683.s002.tif]
